# iDASH secure genome analysis competition 2018: blockchain genomic data access logging, homomorphic encryption on GWAS, and DNA segment searching

**DOI:** 10.1186/s12920-020-0715-0

**Published:** 2020-07-21

**Authors:** Tsung-Ting Kuo, Xiaoqian Jiang, Haixu Tang, XiaoFeng Wang, Tyler Bath, Diyue Bu, Lei Wang, Arif Harmanci, Shaojie Zhang, Degui Zhi, Heidi J. Sofia, Lucila Ohno-Machado

**Affiliations:** 1 UCSD Health Department of Biomedical Informatics, University of California San Diego, La Jolla, CA 92093 USA; 2 School of Biomedical Informatics, The University of Texas Health Science Center, Houston, TX 77030 USA; 3 School of Informatics, Computing and Engineering, Indiana University Bloomington, Bloomington, IN 47408 USA; 4 Department of Computer Science, University of Southern Florida, Orlando, FL 32816 USA; 5 National Human Genome Research Institute, National Institutes of Health, Bethesda, MD 20892 USA; 6 Division of Health Services Research & Development, VA San Diego Healthcare System, San Diego, CA 92161 USA

## Overview

Genome privacy is a twenty-first century challenge that has received relatively low publicity relative to risk, especially when compared to privacy issues surrounding social media or electronic health records [1–3]. This low profile is misleading because (1) the consequences of privacy breaches can be as ominous as those of other data types, and more extensive, since they can affect blood relatives; and (2) the ability for a motivated individual, a particular group, or a nation’s government to conduct an effective attack has increased sharply in the past few years due to improvements in technology. The research community will benefit in the long run from characterizing privacy risks derived from genome data sharing, as well as developing and applying responsible, cost-effective solutions to mitigate these risks. The scientific community must be the first to recognize that, if biometrics such as fingerprints, iris or retinal images, and portraits are considered identifying information and thus redacted from publicly shared datasets, so should be genomes, exomes, and many other downstream data such as transcriptomes, proteomes, etc. It is important to understand that once genomes and related information are made accessible and thus linkable to other data, it is impossible to control what type of inferences can be obtained and what type of sensitive information can be inadvertently disclosed. On the other hand, it is possible to quantify risk and provide commensurate protections for data that are made available for research. Moreover, it is possible to engage the privacy technology community around the theme of responsible genomic data sharing. A growing community of genome privacy researchers has emerged in the past decade. Our purpose is to test the limits of technology that protects genome privacy, while promoting the development of practical strategies that control the risk but preserve the utility of the data as much as possible. The goal is to be proactive, as oppose to wait for a major scandal to set the whole community back for decades, potentially erasing decades of progress in genome analysis and scientific discoveries.

The 5th iDASH Secure Genome Analysis Competition [4] was co-organized in 2018 by the University of California San Diego (UCSD), the University of Texas Science Center (UT Health) and Indiana University Bloomington. Continuing the success of past competitions, our aim was to scaling the protection of the security and privacy of analyses on increasingly large genomic datasets. Specifically, we focused on bridging theory and practice of computational algorithms via community participation. In 2018, we devised three competition tracks, which included (1) blockchain-based genomic dataset access logging (Track 1), (2) secure homomorphic encryption on Genome Wide Association Studies (Track 2), and (3) secure DNA segment searching (Track 3). These three tracks attracted 64 registered teams from 17 countries across America, Europe, and Asia. After 4.5 months of development, 17 teams submitted their solutions by the deadline. We evaluated the submissions in 1 month, using approximately 100 Virtual Machines (VMs). The team from Yale University won Track 1, a joint team from UT Health and UCSD and a joint team from Duality Technologies and Dana Farber Cancer Institute co-won Track 2. A joint team from Microsoft Research and Massachusetts Institute of Technology as well as a joint team from CNRS, ISAE and UQAM co-won Track 3. This special issue of BMC Medical Genomics highlights some most advanced methods and techniques reported during the competition for the three tracks.

## Track 1: Blockchain-based immutable logging and querying for cross-site genomic dataset access audit trail

### Introduction

**Fig. 1:**
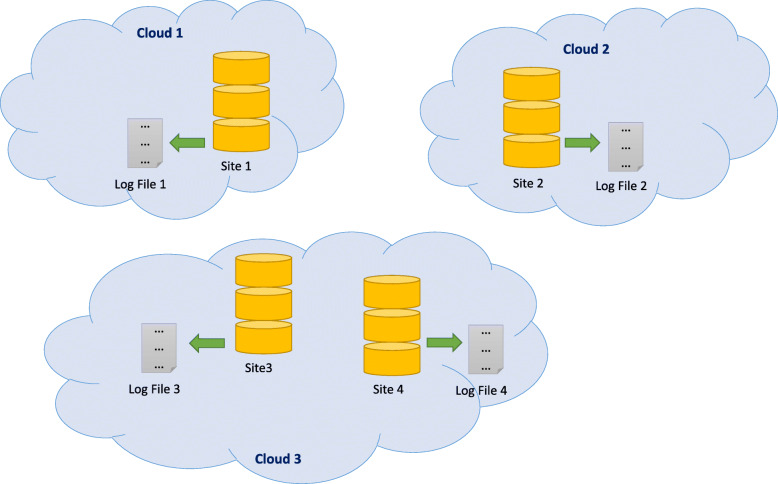
A local logging system. The local logs are managed by each genomic data repository. Cross-site data access behaviors can hardly be detected due to the lack of a global logging system [5]

Auditing data access behavior on genomic data repositories, such as GTEx, is needed because the mismatching of the proposed and the actual data usage should be recognized to avoid research misconduct. For example, if user *X* claimed to use dataset *Y* for analysis *Z* in an institutional review board (IRB) protocol or a data usage agreement (DUA) and actually performed analysis *Z’* on *Y*, this behavior should be identified during the audit process. Although each genomic data repository may have its own *local* logging system, there is currently no *global* logging system to oversee the cross-site data access behaviors (Fig. 1). Intuitively, one can construct a *centralized* global logging system to collect the access logs from each repository. However, such a centralized logging server presents risks such as mutability (i.e., the records may be changed on the central server) and single-point-of-failure (i.e., the global logging system stops working if the central server is under maintenance or being attacked). Additionally, the logging process is not transparent, the interoperability is challenging, and credibility can be questioned.

### Threat model considered in this track

**Fig. 2:**
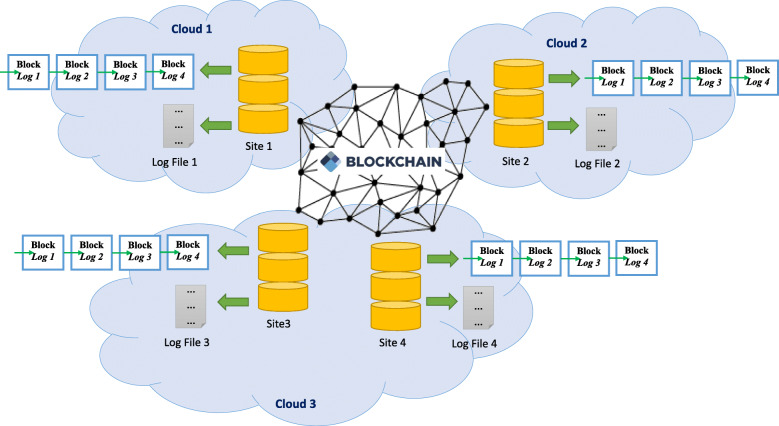
A global logging system. The system is based on blockchain technology, which is managed in a decentralized way [5]

Among various types of potential weaknesses mentioned above, the biggest threat comes from the modification of the genomic data access log when the centralized logging server is compromised and the root privilege is obtained by an attacker. The data misuse records could be eliminated without being noticed, and furthermore, the fake records could also be created to frame a researcher. This is even more critical for the data sets from sensitive populations (e.g., HIV+ patients) where the data is especially valuable and requires additional protection. In this case, technology might be desirable to gain trust and avoid such a threat. Therefore, we propose to adopt *blockchain* and build a *decentralized* global logging system (Fig. 2). Blockchain is the distributed ledger technology that laid the foundation of crypto-currencies, and has been proposed for various genomic/healthcare/biomedical applications [6–12]. Furthermore, by having immutability without a single-point-of-failure, blockchain technology provides benefits such as transparency, interoperability, and credibility. Moreover, each repository can still record access behaviors using traditional log files in parallel to the global logging system. By using the peer-to-peer blockchain as the infrastructure of the logging system, we can prevent the central server attacking threat because (1) there is no single central server to be attacked, and (2) all data usage logs are recorded in an immutable, transparent, and provenance-ensured way.

Although blockchain technology may be a feasible solution, the speed, space and scalability of this new technology are still under investigation for many real-world applications. Also, most of the genomic blockchain applications are still in the proposal phase [5], and the practical aspects for implementations are yet to be studied. Finally, the metrics and methods for evaluating blockchain systems on genomics data are still emerging. To investigate these issues, we developed a new track for the competition. Anticipating a possible use of blockchain technology for retrieval of genomics data, we aimed at understanding to what extent blockchain may be applied to serve as a global logging system. As such, the goal of this competition is to develop blockchain-based ledgering solutions to log and query the user activities of accessing genomic datasets (e.g., GTEx) across multiple sites.

### Data and sub-tasks

The datasets were generated using a software we developed to simulate genomic data access behaviors. We assume multiple users’ simultaneous access of various types of resources (i.e. genomic datasets) on multiple sites. Each user has the following ordered behavior: request to access the resource, view the resource, access the resource, and, optionally, the user may receive a risk score derived from privacy protection algorithms. We used a simulator to generate both training and test data, which were log files containing records (transactions) such as “at 2018-08-13 08:21:43, user 10 viewed resource 3 on Site 1”. The *training* data we provided to the participating teams included four data access log files, representing user access activities from four sites. Each log file contained 100,000 records. The *test* data were not provided to the participating teams. We generated three datasets with different sizes (*small* = 50,000, *medium* = 100,000, and *large* = 200,000 records per site) to test the scalability of the solutions. We also increased the number of parameters and types of resources to encourage more generalizable solutions.

**Fig. 3:**
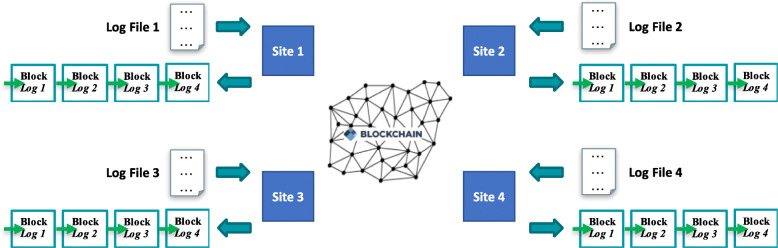
Logging sub-task of our blockchain competition. Sites post all transactions to the chain [5]

**Fig. 4:**
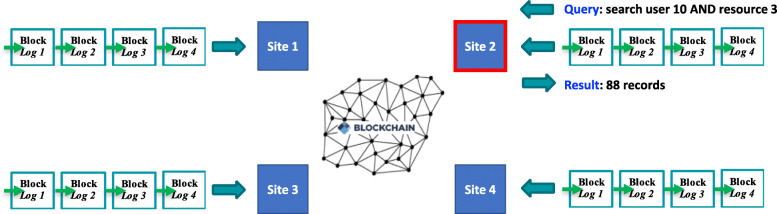
Querying sub-task of our blockchain competition. Site2 queries the chain using Boolean logic, and ranks results (any site can query the chain as there is transparency across the sites) [5]

There were two sub-tasks of the blockchain competition: logging and querying. For the logging sub-task (Fig. 3), the solution was required to store all user access log records on-chain, while storing no records off-chain. For the querying sub-task (Fig. 4), the solution needed to allow a user to search using any field of one log line (e.g., User_ID), use any “AND” combination (e.g. User_ID AND Resource_ID), sort the results (e.g. ascending/descending order), and query the data from any of the four sites.

### Evaluation and test queries

To evaluate the participating teams, our criteria included (1) accurately log/query results using the test data, and (2) high performance in speed, storage/memory cost, and scalability. For the first criterion, the log and query results should be 100% accurate in order to be considered a valid solution. For the second criterion, the order of importance was speed > storage/memory cost > scalability.

To test the record queries, we generated 50 distinct search queries to test the solutions, including 12 single-line-type test queries (e.g. search for a specific record in each of the four log files), 26 column-type test queries (e.g. search for all records related to a specific resource), and 12 combo-type test queries (e.g. search for all records related to a specific resource AND a specific user). We also provided 4 example queries and solutions with the training data with correct answers for the participating teams to verify their solutions.

The process to apply our evaluation criteria and to run the test queries on the test data was as follows. First, we ran the software of each participating team using the small, medium and large test datasets, and measured the following four metrics:

- *Insertion* (in seconds): the maximum time of insertion plus synchronization (records are visible on all 4 nodes), confirmed by using a query to check the results, with a timeout limit of 70 h.
- *Query* (in seconds): the average time for 50 test queries.
- *Storage* (in GB): the difference of disk usage before and after the insertion process.
- *Memory* (in MB): the maximum usage for the insertion process.

Next, we normalized each metric to a *raw score* from 0 to 100 among all teams. These raw scores are then weighted-summed to a *subtotal score* for each of the test datasets (i.e. small, medium and large) with weights of 35% for Insertion, 35% Query, 15% Disk, and 15% Memory. Finally, to take scalability into account, we computed a weighted average of the subtotal scores by the number of records (i.e. small = 50,000, medium = 100,000, and large = 200,000) to an *overall score* (from 0 to 100) for final ranking.

### Blockchain platform and test environment

Based on a recent review of popular blockchain platforms [7], we chose to use MultiChain (a fork of the Bitcoin Blockchain) [13, 14], which can reach about 1000 maximum transactions per second. We provided each participating team with 4 VMs, and each had 2-Core CPU, 8GB RAM and 100GB storage, with a 64-bit Ubuntu 14.04 operating system. We utilized 64 VMs on the Google Cloud Platform [15] for testing and evaluation.

### Participating teams and results

Seven teams completed the competition. Their names and affiliations are as follows (in alphabetical order): BlockchainProvenance (UT Dallas), CSI-Lab (Rutgers University), GersteinLab (Yale University), JUICE (Wuhan University, Juzix), Sandia (Sandia National Laboratories), SUCloud (Syracuse University), and YCao31 (Emory University, University of Central Florida, and Kyoto University).

**Fig. 5:**
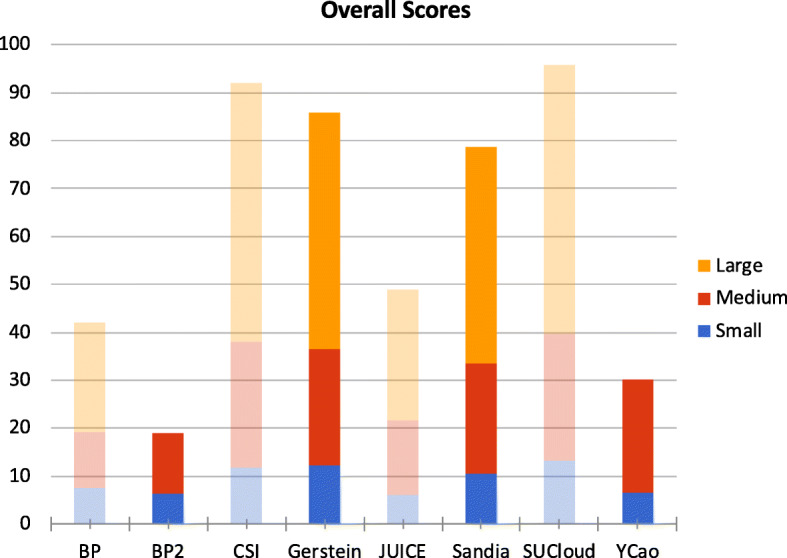
Querying sub-task of our blockchain competition. *BP* = BlockchainProvenance, *CSI* = CSI-Lab, *Gerstein* = Gerstein-Lab, *YCao* = Ycao31. Higher scores indicate better performance. A bar is dimmed if the results of any test dataset were not accurate; in this case the corresponding team was not included in the final ranking. For *BP2* and *YCao*, the results for the small and medium test datasets were accurate, however their solutions were not able to complete the insertion of the large test dataset, therefore their subtotal scores for the large test dataset were both set to zero

The overall scores are summarized in Fig. 5, and the detailed measurements on the small, medium and large test datasets are shown in Tables 1, 2, and 3, respectively. There were 8 submissions from the 7 participating teams. BlockchainProvenance had an additional submission shortly after the deadline, which we graded for the sake of completion, and therefore is included only for reference purposes. This is referenced as BP2 applied various techn2 in all figures and tables. All submissions successfully completed the insertion sub-task for the small and medium test datasets, and 6 of them finished inserting records to the large test dataset within 70 h. There were 4 submissions that generated accurate query results for the small and medium test datasets and, among them, only 2 submissions also showed accurate results in the large test dataset. These 2 submissions (GersteinLab and Sandia) also demonstrated nearly linear scalability for all measurements.

**Table 1:** Measurements on the small test dataset (50,000 records)

Small	Complete	Accurate	Insertion	Query	Disk	Memory
**BP**	Yes	No	02:36:10	00:00:12	1.570 GB	41 MB
BP2*	Yes	Yes	03:53:50	**00:00:02**	1.670 GB	42 MB
**CSI**	Yes	No	00:42:45	00:00:13	1.252 GB	10 MB
**Gerstein**	Yes	Yes	**00:12:30**	00:00:28	**0.400 GB**	**18 MB**
**JUICE**	Yes	No	01:03:54	00:02:13	2.300 GB	6 MB
**Sandia**	Yes	Yes	00:43:09	00:00:26	1.438 GB	22 MB
**SUCloud**	Yes	No	00:06:28	00:00:15	0.307 GB	15 MB
**YCao**	Yes	Yes	04:24:20	00:00:08	1.458 GB	26 MB

*Complete* indicates whether the insertion process is completed within 70 h, and *Accurate* indicates whether the results of all test queries are accurate. A cell is underscored if the results are not accurate. The **bold** cells of measurements indicate the best performances among accurate results. The asterisk (*) indicates submissions received shortly after the deadline

**Table 2:** Measurements on the medium test dataset (100,000 records). The notation is the same as the one used in Table 1

Medium	Complete	Accurate	Insertion	Query	Disk	Memory
**BP**	Yes	No	19:18:11	00:00:47	7.870 GB	53 MB
BP2*	Yes	Yes	28:50:30	**00:00:04**	3.270 GB	54 MB
**CSI**	Yes	No	01:27:51	00:00:27	1.652 GB	10 MB
**Gerstein**	Yes	Yes	**00:22:56**	00:00:54	**0.700 GB**	30 MB
**JUICE**	Yes	No	02:04:17	00:04:16	4.300 GB	5 MB
**Sandia**	Yes	Yes	01:23:51	00:00:47	2.695 GB	**28 MB**
**SUCloud**	Yes	No	00:12:34	00:00:30	0.552 GB	15 MB
**YCao**	Yes	Yes	03:05:33	00:00:16	2.558 GB	32 MB

**Table 3:** Measurements on the large test dataset (200,000 records). The notation is the same as Table 1. *BP2* and *YCao* did not complete the insertion process within the time limit (70 h)

Large	Complete	Accurate	Insertion	Query	Disk	Memory
**BP**	Yes	No	66:27:49	00:01:00	5.570 GB	78 MB
BP2*	No	No	–	–	–	–
**CSI**	Yes	No	02:52:13	00:01:10	2.452 GB	10 MB
**Gerstein**	Yes	Yes	**00:55:46**	**00:02:11**	**1.300 GB**	54 MB
**JUICE**	Yes	No	04:39:10	00:12:56	7.800 GB	6 MB
**Sandia**	Yes	Yes	04:07:17	00:01:48	5.438 GB	**41 MB**
**SUCloud**	Yes	No	00:27:43	00:01:05	1.227 GB	15 MB
**YCao**	No	No	–	–	–	–

Based on our evaluation criteria, the final winning teams were GersteinLab (first place), Sandia (second place), and YCao31 (third place). The research papers from the winning teams describing their approaches are included in this special issue [16–18]. GersteinLab created a data frame from the blockchain to allow efficient queries [16]. Sandia employed a two-level indexing method to support efficient queries with single clause constraints [17]. YCao31 designed a hierarchical structure to support efficient range queries on the timestamp field [18]. Another team BP/BP2 also described the solution in the research paper included in this special issue [19]. BP/BP2 applied various techniques and optimizations (e.g., bucketization, simple data duplication and batch loading) to speed up their solution [19]. The method is very innovative to address the logging/querying challenge of the competition.

### Summary

Based on the outcomes of the submissions, using blockchain technology to support a global and immutable logging system is feasible. The best solution was able to store 800,000 genomic dataset access records (i.e., 200,000 from 4 sites) within 1 h and query them accurately within 3 min. It can scale almost linearly in terms of insertion time, query time, storage usage, and memory usage. Although there is still room for an improvement in efficiency, this reasonable performance showed the potential of adopting an immutable, decentralized, transparent, interoperable and credible ledger for genome data access transactions.

## Track 2: Secure parallel genome wide association studies using homomorphic encryption

### Introduction

As more human genomic data are generated, there is a growing trend to use cloud computing services to store and analyze these data for scalability and cost-effectiveness. When outsourcing the human genome data to a third-party cloud service provider, there can be concerns about privacy risks and the practicability of communication. This task is developed to challenge participating teams in coming up with a secure outsourcing solution to compute a Genome Wide Association Study (GWAS) based on homomorphically encrypted data. Fully homomorphic encryption (FHE) under the Ring Learn With Error (RLWE) framework is considered post-quantum safe and does not require communication once encrypted data are provisioned on a service provider. The basic idea of homo-(same) morphic-(shape) encryption is to convert plaintext data into encrypted data satisfying certain mathematical properties (i.e., structural preserving mapping in the algebraic definition) so that the computation on the encrypted data have one-to-one mapping to the corresponding operations in the plaintext, therefore, the results are kept consistent (after decryption). Data owners only need to make a one-time encryption (using public key) and deposit encrypted data onto the cloud. Then, they can execute different algorithms to obtain encrypted results (which can be decrypted with the secret key). The entire process is secure without leaking any information.

Based on the previous success in building a learning model (i.e., logistic regression) on encrypted data (Track 3: Homomorphic encryption (HME) based logistic regression model learning in 2017) [20], we came up with a more challenging task for 2018. That is, given encrypted genome data that contain thousands of Single Nucleotide Polymorphisms (SNPs) and hundreds of samples, participants are asked to outsource the storage and computation on a third-party server to carry out GWAS (based on linear or binary logistic regression) to compute *p*-values of different SNPs.

We followed the additive genetic model using the Cochran-Armitage trend test [21], which is equivalent to the score test in the logistic regression [22]. The model for individual SNPs was tested using the following:

$$ \mathit{\log}\left({p^i}_j/1-{p^i}_j\right)=1+{\beta}_{\mathbf{1}}{X}^i+{\beta}_2{Z^i}_j $$

where *p*^*i*^_*j*_ = *P*(*Y*^*i*^ = 1) correspond to the probability that individual *i* has the disease or not. The model has two sets of parameters: *β*_**1**_ corresponds to the parameters for the covariants (e.g., demographics, pre-conditions) and *β*_**2**_ corresponds to a single parameter for an individual SNP *j*. The test is to check whether *β*_**2**_ equals zero or not, which indicates whether the disease *Y*^*i*^ has a dependence on SNP *j*.

**Fig. 6:**
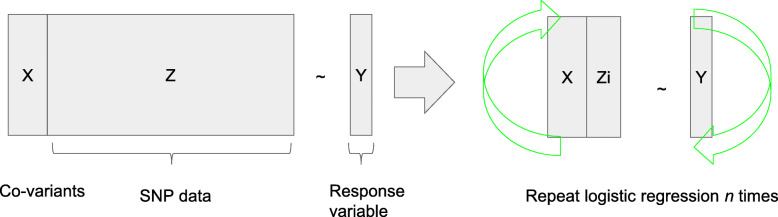
Traditional Genome Wide Association Study (GWAS) scans the entire list of SNPs iteratively. But such an approach is not computationally practical for fully homomorphic encryption

Although significant performance improvements over existing solutions have been demonstrated for constructing logistic regression on encrypted data, it is still quite computationally intensive (i.e., ~ 10 min per SNP based on the best entry in iDASH 2017). Direct implementation of linear or logistic regression based GWAS would require building one model for each SNP, which makes it technically impractical when we need to deal with millions of SNPs (see Fig. 6).

In order to come up with a task that is feasible, considering the performance limitation of FHE, we carefully studied the literature and identified an alternative semi-parallel algorithm [23] for this competition, which relies on an approximation to reduce the necessary rounds of computation. The main idea is to assume the parameters for covariants will stay nearly the same for all SNPs and convert *n* logistic regressions into one for covariants, followed by another single-step parallelizable regression on all SNPs. The challenge here is to develop efficient packing and parallelization algorithms to make full use of the memory and computation resources.

### Threat model considered in this track

We are considering the outsourcing security in this track, which include both data security and model security. Our scenario applies to situation when owners of the data want to outsource them on an untrusted cloud computing environment. The threat model here involves include information hijacking, system hacking, malicious hosts, and other types of inference attack. We count information leakage during the information exchange, data storage, model construction and evaluation. We also consider the potential side channel attacks like monitoring CPU or memory usage, looking over page fault patterns, communication bandwidth, etc.

### Data and compute environment

We prepared training data extracted from the Personal Genome Project [24]. The training data have 3 covariates (age, weight, height), 10,643 SNPs (all binary indicating minor or major alleles), and 1 outcome variable for a total of 245 individuals, partitioned into two groups by the presence of high cholesterol, 137 for control group and 108 for the disease group. The reserved test data are on the same population (3 covariates + 14,841 SNPs), which represent the same 245 individuals (therefore, the same covariants) and different SNPs.

We asked the teams to develop solutions that have at least 128 bits of security, following recommended parameters from the Homomorphic Encryption Standardization Workshop security whitepaper [25], and the solutions need to be strictly non-interactive. Our evaluation is based on the following measures: model accuracy, round-trip time, and memory consumption, which are prioritized in the given order (accuracy > time > memory). Our evaluation environment is provided by the School of Biomedical Informatics at UT Health, with 25 VMs that are equivalent to Amazon T2 Xlarge instances, which have 4 vCPU, 16GB memory, disk size around 200GB.

### Evaluation results

We received 13 solutions, submitted from 7 teams by the deadline. The research papers from the participating teams to describe the methods are included in this special issue [26–30]. Table 4 summarizes the results, including memory consumption, round-trip time cost, and F1 scores at different cutoffs. In terms of model accuracy, there is no statistical significance for the first 4 teams, while UCSD and Duality show a clear advantage on running time and peak memory usage when compared with other competing entries. Because the difference in F1 score is relatively small, we also conducted an additional test on the differences between Area Under the ROC curve (AUC) to ensure the robustness of our comparison (AUC compares the entire curve instead of a single point for F1). We used DeLong’s Test [31] for AUCs and randomly sampled 1499 SNPs (~ 10%) to construct ROC curves, repeating 10 times to obtain mean and standard deviation. Note that we transformed semi-parallel GWAS outputs (estimated probabilities) to 0–1 labels according to *p*-value cutoff 1E-2 and 1E-5 in the experiments, which are illustrated in Table 5.

**Table 4:** Overall results as in terms of end-to-end time/memory costs and F1 scores at different cutoffs. Note that gold and semi refer to the original GWAS results and semi-parallel GWAS results in plaintext, respectively. We expect teams will benefit from using our suggested semi-parallel algorithm to design their fully homomorphic encrypted counterpart, but we encouraged solutions to approximate the gold standard (plaintext original GWAS model) as much as possible to be useful in practice

Team	Submission	Schemes	End to End Performance		Evaluation result (F1- Score) at different cutoffs							
			Running time (mins)	Peak Memory (M)	0.01		0.001		0.0001		0.00001	
					Gold	Semi	Gold	Semi	Gold	Semi	Gold	Semi
A*FHE	A*FHE −1 +	HEAAN	922.48	3777	0.977	0.999	0.986	0.999	0.985	0.999	0.966	0.998
	A*FHE −2		1632.97	4093	0.882	0.905	0.863	0.877	0.827	0.843	0.792	0.826
Chimera	Version 1 +	TFHE & HEAAN (Chimera)	201.73	10,375	0.979	0.993	0.987	0.991	0.988	0.989	0.982	0.974
	Version 2		215.95	15,166	0.339	0.35	0.305	0.309	0.271	0.276	0.239	0.253
Delft Blue	Delft Blue	HEAAN	1844.82	10,814	0.965	0.969	0.956	0.944	0.951	0.935	0.884	0.849
UC San Diego	Logistic Regr +	HEAAN	1.66	14,901	0.983	0.993	0.993	0.987	0.991	0.989	0.995	0.967
	Linear Regr		0.42	3387	0.982	0.989	0.980	0.971	0.982	0.968	0.925	0.89
Duality Inc	Logistic Regr +	CKKS (Aka HEAAN), pkg.: PALISADE	3.8	10,230	0.982	0.993	0.991	0.993	0.993	0.991	0.990	0.973
	Chi2 test		0.09	1512	0.968	0.983	0.981	0.985	0.980	0.985	0.939	0.962
Seoul National University	SNU-1	HEAAN	52.49	15,204	0.975	0.984	0.976	0.973	0.975	0.969	0.932	0.905
	SNU-2		52.37	15,177	0.976	0.988	0.979	0.975	0.974	0.969	0.939	0.909
IBM	IBM-Complex	CKKS (Aka HEAAN), pkg.: HElIb	23.35	8651	0.913	0.911	0.169	0.188	0.067	0.077	0.053	0.06
	IBM- Real		52.65	15,613	0.542	0.526	0.279	0.28	0.241	0.255	0.218	0.229

+ no statistical significance in terms of discrimination=

**Table 5:** Statistical tests on AUCs using DeLong’s Test, showing that there is no statistical difference among the top four teams in the previous table at a significance level of 0.01 for both cutoff thresholds (1E-2 for the triangle above the main diagonal of the matrices, and 1E-5 for the triangle below the main diagonal of the matrices) for converting the outputs of semi-parallel GWAS (estimated probabilities) to binary labels (for AUC computation)

	Duality lr	UCSD-1	A*FHE-1	Chimera-1
**Duality lr**		1.0000 ± 0.0000	0.3400 ± 0.0935	0.3121 ± 0.1332
**UCSD-1**	0.8959 ± 0.2195		0.3400 ± 0.0934	0.3156 ± 0.1332
A*FHE-1	0.7072 ± 0.3117	0.8113 ± 0.3043		0.4090 ± 0.3383
**Chimera-1**	0.2606 ± 0.1306	0.2632 ± 0.1296	0.2044 ± 0.0793	

**Fig. 7:**
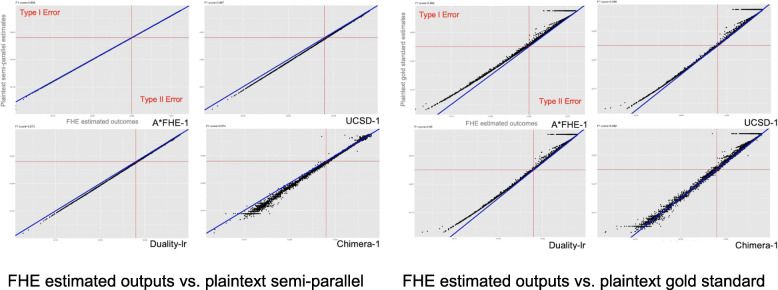
Visualized comparison of top 4 models against plaintext semi-parallel outputs and plaintext gold standard estimates. The best model is expected to be perfectly aligned with the blue curve

The UCSD team used a sparse binary secret setting that is compatible with the security setting but not listed in the white paper. Their adjusted performance using the white paper setting is close to that of Duality (but not listed here as it is after competition), so Duality and UCSD were co-winners of the competition. The Chimera team is a runner-up. We also decomposed the time and memory cost in terms of different phases, including key generation, encryption, computing and decryption (Table 6). In Fig. 7, we illustrate the differences between the top teams FHE estimated outputs (after decryption) against their plaintext counterpart (semi-parallel) and the plaintext gold standard.

**Table 6:** Detailed decomposition of time and memory cost at different stages of the computation

Team	Submission	Time consumption and memory cost*							
		KeyGeneration		Encryption		Computing		Decryption	
		Time (MIN)	Memory (MB)	Time (MIN)	Memory (MB)	Time (MIN)	Memory (MB)	Time (MIN)	Memory (MB)
A*FHE	A*FHE −1			0.79	822	921.25	3777	0.44	410
	A*FHE −2			1.12	528	1631.01	4093	0.84	
Chimera	Version 1	0.30	10,375	8.92	10,375	192.80	9444	0.02	8
	Version 2		13,658	7.93	10,358	208.00	15,166	0.02	73
Delft Blue	Delft Blue			4.42	4723	1840.40	10,814	< 0.01	10,814
UC San Diego	Logistic Regr	0.95	5205	0.34	8222	1.33	14,901	< 0.01	
	Linear Regr	0.01	246	0.38	3095	0.04	3387	< 0.01	
Duality Inc	Logistic Regr	0.35	5211	0.34	8953	3.46	10,230	< 0.01	10,228
	Chi2 test	< 0.01	283	0.05	1045	0.05	1512	< 0.01	1413
Seoul National University	SNU-1		1928	6.17	6584	45.40	15,204	0.92	15,204
	SNU-2	1.65	58	1.28	6525	50.45	15,117	0.63	
IBM	IBM-Complex			3.30		19.10	8651	0.95	
	IBM- Real			6.20		45.50	15,613	0.95	

*the memory usage refers to the peak memory of the corresponding operation, all team's process fit in the 16GB environment

### Summary

Track 2 breaks the previous record in FHE computation and shows that a practical secure protocol for basic genome data analysis like GWAS is possible. High accurate secure computation for 15 k SNPs can be accomplished within 2 min. Another interesting observation is that all competition solutions choose a common encryption framework CKKS/HEAAN [32], compared to several different frameworks in the previous year. This suggests a community “consensus” that certain strategies like the approximate arithmetic homomorphic encryption are amenable to numerical optimization for problems such as the one we presented, and solutions that involve machine learning and statistical testing.

## Track 3: Secure search of DNA segments in large genome databases

### Introduction

Databases of whole genome-scale genotypes are becoming available in public (e.g., the UK Biobank [33]) or private (e.g., from direct-to-consumer genetic testing providers [34], such as 23andMe [35]) databases. One of the straightforward applications for these data is to identify relatives, in particular distant relatives, which has not only been useful for ancestry and genealogy analysis by academic researchers, citizen scientists and individual consumers [34], but also has strong implications for forensics (a great example is the arrest of the alleged Golden State serial killer in California using genetic genealogy methods [36]).

Many algorithms have been developed for kin relationship inference from genotype data. Unlike the close genealogical relatives such as parent and siblings that can be derived from the genotypes on multiple genetic marker loci (e.g., microsatellites), inference for distant relatives often require genome-wide genotype data, and thus may be computationally intensive [37, 38], especially when one is searching a query genome against a large genome database (with thousands to millions of genomes).

### Threat model considered in this track

Searching these large databases for matched DNA segments (i.e., consecutive variant matches) represents an efficient approach to the identification of distant relatives. However, privacy concerns must be addressed before a practical querying system can be adopted. Here, we assume both the querier and the database owner are semi-honest (hosnest but curious), and may attempt to learn the private genomic information owned by the other party. To protect the query genome as well as the genomes in the database, we hope to encrypt the query and database genomes, respectively, while allowing their comparison using ciphertext. Specifically, we designed this track to challenge participating teams for developing a secure two-party computation solution to identify *set-maximal matches* (longer than a given threshold) [39] between a query genome and a large database of genotypes, *X* = {*x*_*1*_, *x*_*2*_, …, *x*_*M*_}, where *x*_*i*_ represents the vector of genotypes (herein, we consider only the *single nucleotide variants*, or SNVs) for the i-th genome, *S,* in which the SNVs are sorted with respect to their locations on the genome.

Formally, given *X* and a query genotype set *z*, a list of consecutive SNVs indices between scalars *a* and *b* (S [a,b]; a < b) is *set-maximal* if

1. There is a *x*_*i*_*∈ X* such that the genotypes of *x*_*i*_ and *z* match exactly: *x*_*i,k*_ = z_*k*_ for *k ∈* [a,b];
2. S [a,b] cannot be extended without genotype mismatches between *x*_*i*_ and *z*, i.e., *x*_*i,a-1*_ *≠ z*_*a-1*_ or *x*_*i,b + 1*_ *≠ z*_*b + 1*_*;*
3. There is no *x*_*j*_*∈ X* (*j ≠ i*) such that the genotypes of *z* and *x*_*j*_ exactly match.

Note that a secure algorithm based on homomorphic encryption has been previously proposed [40]. However, it introduced high computation overhead. Here, we want to challenge the community to develop novel, efficient and secure two-party computation algorithms to identify whether genome *x* in database *X* exhibits the set-maximal match longer than a given threshold *L* with a query genome *z* such that *z* is not exposed to the owner of the database while no genome in *X* is exposed to the querier.

### Data and evaluation criteria

The participating teams were given a database of 1000 human genomes in the format of haplotype SNV sequences (supposed to be hosted by a *data owner*), and a query genome of the same format (supposed to be submitted by a data *querier*), and were then challenged to design a secure two-party algorithm between the owner and the querier to compute all set-maximal matches longer than a given threshold of *1000* SNV sites between the query genome and the genomes in the database, without exposing the database to the *querier* or exposing the query genome to the *owner.* Here, we assume that the database genomes do not contain errors; as a result, the output genomic segments represent perfect matches between the corresponding database and query genomes. For testing purposes, we provide a database of 1000 genomic sequences (each consisting of 100,000 SNVs in VCF format), a few sample queries, as well as the expected output from these queries.

Each team is required to submit their non-interactive solution in executable forms (and source code if possible). Each team can choose to preprocess (e.g., index) the database, which can be implemented in a non-secure algorithm. In this case, the preprocessing algorithm and the querying algorithm (for secure two-party computation) should be implemented and submitted separately. We evaluated the submitted solutions using datasets of similar size, but different from the provided data to test the performance of the submitted solutions. We checked the security compliance (at least 128-bit security level), accuracy of the results (expected to be the same or very close to the results of the non-secure algorithm), speed (database preprocessing time and query time are considered separately) as well as the size of the transferred data.

### Participating teams, results and summary

Three teams submitted their solutions to the task: CNRS (France), Microsoft Research (USA), and University of Tsukuba and Riken (Japan). The CNRS team submitted two solutions, one fast algorithm and one accurate (but slower) algorithm. Unfortunately, none of the submitted solutions were able to report accurate results in most testing cases with the query genomes of 100,000 SNV sites as the intended input of the challenge. When tested on a smaller input size (consisting of 100 SNV sites), the accurate solution of CNRS can report 23 correctly matched genomes out of 26 test cases, while Microsoft Research’s solution reported correctly 12 out of 26 cases and the fast solution of CNRS reported correctly 4 out of 26 cases. Microsoft Research’s solution can complete the task in 7 s, while the CNRS accurate solution takes much more time (26 h) and the CNRS fast solution takes 5 h. Based on these results, CNRS and Microsoft Research shared the winning award. We conclude more efficient algorithms are needed to tackle this challenging task. A research paper from the Microsoft team that describes their approach is included in this special issue [41].

## Conclusions

Understanding genome privacy risks and developing practical solutions is challenging. Our competition attracted teams from different countries to address significant issues in genome data sharing via privacy technology. The new methods and techniques illustrated some important advances in this year’s competition, and revealed new and promising results for practical biomedical privacy research. For Track 1, we showed that a blockchain-based immutable logging system is feasible with observed storage of 800 K genomic dataset access records from 4 sites within 1 h, and a query time within 3 min. For Track 2, we concluded that a practical secure protocol for basic genome data analysis is possible for high accuracy secure GWAS for 15 k SNPs, within 2 min. For Track 3, we showed that complicated protocols such as secure multiparty ancestry analysis are viable, although further research is still needed for fast and scalable approaches. These findings can contribute to enhance genomic security and privacy by bringing together innovations from the scientific community that challenge current practices.

## Data Availability

We have created datasets that are available on http://www.humangenomeprivacy.org/2018/ under each track for share with the participating teams. The code was not shared for every track because some participants were from companies. We are planning to request code sharing for the upcoming competitions.

## Abbreviations

AUC: Area Under the receiver operating characteristic Curve
CPU: Central Processing Unit
DNA: DeoxyriboNucleic Acid
DUA: Data Usage Agreement
FHE: Fully homomorphic encryption
GTEx: Genotype-Tissue Expression
GWAS: Genome Wide Association Study
HME: HomoMorphic Encryption
iDASH: integrating Data for Analysis, anonymization, and SHaring
IRB: Institutional Review Board
RAM: Random-Access Memory
RLWE: Ring Learn With Error
SNP: Single Nucleotide Polymorphism
SNV: Single Nucleotide Variant
VCF: Variant Call Format
VM: Virtual Machine
